# Identification of Candidate Biomarkers and Prognostic Analysis in Colorectal Cancer Liver Metastases

**DOI:** 10.3389/fonc.2021.652354

**Published:** 2021-08-04

**Authors:** Tianhao Zhang, Kaitao Yuan, Yingzhao Wang, Mingze Xu, Shirong Cai, Chuangqi Chen, Jinping Ma

**Affiliations:** Division of Gastrointestinal Surgery Center, The First Affiliated Hospital, Sun Yat-sen University, Guangzhou, China

**Keywords:** colorectal cancer, differentially expressed genes, liver metastasis, biomarkers, prognostic analysis

## Abstract

**Background:**

Colorectal cancer (CRC), one of the most common malignant tumors worldwide, has a high mortality rate, especially for patients with CRC liver metastasis (CLM). However, CLM pathogenesis remains unclear.

**Methods:**

We integrated multiple cohort datasets and databases to clarify and verify potential key candidate biomarkers and signal transduction pathways in CLM. GEO2R, DAVID 6.8, ImageGP, STRING, UALCAN, ONCOMINE, THE HUMAN PROTEIN ATLAS, GEPIA 2.0, cBioPortal, TIMER 2.0, DRUGSURV, CRN, GSEA 4.0.3, FUNRICH 3.1.3 and R 4.0.3 were utilized in this study.

**Results:**

Sixty-three pairs of matched colorectal primary cancer and liver metastatic gene expression profiles were screened from three gene expression profiles (GSE6988, GSE14297 and GSE81558). Thirty-one up-regulated genes and four down-regulated genes were identified from these three gene expression profiles and verified by another gene expression profiles (GSE 49355) and TCGA database. Two pathways (IGFBP-IGF signaling pathway and complement-coagulation cascade), eighteen key differentially expressed genes (DEGs), six hub genes (*SPARCL1*, *CDH2*, *CP*, *HP*, *TF* and *SERPINA5*) and two biomarkers (*CDH2* and *SPARCL1*) with significantly prognostic values were screened by multi-omics data analysis and verified by Gene Expression Omnibus (GEO) and The Cancer Genome Atlas (TCGA) cohort.

**Conclusions:**

In this study, we identified a robust set of potential candidate biomarkers in CLM, which would provide potential value for early diagnosis and prognosis, and would promote molecular targeting therapy for CRC and CLM.

## Introduction

Colorectal cancer (CRC) is one of the most common malignant tumors worldwide. According to global cancer statistics, more than 1.9 million new CRC cases and 935,000 deaths were reported in 2020, accounting for approximately one-tenth of cancer cases and deaths. Overall, the incidence of CRC ranks third globally, and the mortality rate ranks second (1). Colorectal cancer liver metastasis (CLM) is one of the primary causes of this high mortality rate, which occurs in 30% of CRC patients, accounting for two-thirds of the related deaths (2). Further, more than 50% of patients relapse within 2 years after CLM resection (3).

Numerous clinical data indicate that the liver is the most common target organ for CRC metastasis. To date, the relevant mechanisms underlying the formation and progression of liver metastasis during disease progression of this disease have been extensively studied, however, the pathogenesis has not been fully elucidated. With the rise of emerging technologies such as genome and transcriptome sequencing, gene-editing technology, and artificial intelligence (AI), biomedical research is undergoing revolutionary changes, gradually transforming from traditional medicine to precision medicine. Among them, next-generation sequencing (NGS) technology has revolutionized our ability to obtain information from the genome regarding the DNA sequence itself, as well as the state of the transcriptome and the epigenome (4). However, most NGS technologies have not solved the functional interpretation of differentially expressed genes (DEGs) to identify appropriate key genes. Indeed, the occurrence and development of CLM involves a myriad of epigenetic and genetic changes within multiple functional signaling pathways. These different networks are susceptible to regulation by genetic and epigenetic events, leading to diversity in the expression profiles. Therefore, the combination of integrated bioinformatics methods and expression profiling technology may have the ability to overcome, thus screening suitable biomarkers and guiding the selection of clinical systemic prevention, diagnostic, and treatment options.

In this study, we aimed to analyze and predict candidate biomarkers of CLM. Firstly, key DEGs were screened from gene expression profiles of GEO database (Gene Expression Omnibus). Specifically, we identified the biological functions and signal transduction pathways of the selected DEGs through GO (Gene ontology) and KEGG (Kyoto Encyclopedia of Genes and Genomes) enrichment analysis. Further, we constructed protein-protein interaction (PPI) networks and analyzed prognostic values of candidate genes in CRC through data mining. Finally, to evaluate whether the selected candidate biomarkers are reliable, we analyzed the gene expression profile data sets, RNA-Seq data sets of GEO and TCGA databases, and used CNV (copy number variation), GESA (Gene Set Enrichment Analysis), IHC (immunohistochemistry), and other methods for verification. Moreover, the verification results are basically consistent with our research conclusions. Therefore, this study will contribute to understanding the molecular mechanism in depth and contribute to the discovery of new appropriate molecular diagnostic and therapeutic targets, and more accurately prognose long term outcome in patients with CRC.

## Materials and Methods

### NCBI-GEO

NCBI-GEO (Gene Expression Omnibus) (https://www.ncbi.nlm.nih.gov/geo) is a free microarray/gene profile and NGS database. In our study, we screened GSE6988 (5), GSE14297 (6), and GSE81558 (7) microarray datasets containing expression profiles of matching colorectal liver metastases (primary colon cancer samples and liver metastasis samples of the same patient), and 63 pairs of matching primary CRC and liver metastatic cancer tissues were used. To confirm the reliability of DEGs identified from the three GSE datasets, this study selected the GSE49355 (8) microarray dataset, which includes 13 pairs of matched primary colorectal cancer and liver metastasis samples, in the GEO database for verification.

### GEO2R

GEO2R (https://www.ncbi.nlm.nih.gov/geo/geo2r/), is a data processing tool on GEO. Statistically significant differences were identified based on a classic t-test, considering p < 0.05 and |log FC| > 1 as the cut-off criteria. In this study, we used GEO2R to filter the original data to determine DEGs and visualized them with SANGERBOX (http://sangerbox.com/Index), FUNRICH and R.

### DAVID

Database for Annotation, Visualization and Integrated Discovery (DAVID) 6.8 (https://david.ncifcrf.gov/home.jsp) is a comprehensive, functional annotation website that help investigators better clarify the biological function of submitted genes (9). In our study, the Gene Ontology (GO) enrichment analysis and Kyoto Encyclopedia of Genes and Genomes (KEGG) pathway enrichment analysis of DEGs were isolated from DAVID 6.8 and visualized with EHbio (http://www.ehbio.com/ImageGP/index.php/home/index/scatterplot.html). Biological processes (BP), cellular components (CC) and molecular function (MF) were included in GO enrichment analysis.

### STRING

STRING (https://string-db.org/) aims to collect, score, and integrate all publicly available sources of protein-protein interaction (PPI) data and complement these with computational predictions of potential functions (10). We used STRING to develop and construct DEG-encoded proteins and PPI networks and analyze the interactions among candidate DEG-encoded proteins, and we visualized them with CYTOSCAPE 3.7.2.

### UALCAN

UALCAN (http://ualcan.path.uab.edu/analysis.html), a comprehensive web resource, provides analyses based on The Cancer Genome Atlas (TCGA) and MET500 cohort data (11). In this study, we used UALCAN to analyze the expression profiles and prognostic values of DEGs. Student’s t-test was used to generate a p-value. The p-value cutoff was 0.05.

### ONCOMINE

ONCOMINE (www.oncomine.org), currently the world’s largest oncogene chip database and integrated data mining platform, contains 715 gene expression data sets and data from 86,733 cancer tissues and normal tissues (12). In this study, DEG expression was assessed in CRC tissues relative to its expression in normal tissues, and a p-value of 0.05, a fold change of 1.5, and a gene rank in the top 10% were set as the significance thresholds.

### GEPIA

GEPIA (http://gepia.cancer-pku.cn/index.html) is an analysis tool containing RNA sequence expression data of 9,736 tumors and 8,587 normal tissue samples, which was developed at Peking University (13). In this study, we performed a differential gene expression analysis of tumor and normal tissues, and prognostic analysis of DEGs with the “Expression Analysis” module of GEPIA2. The p-value cut-off was 0.05 Student’s t-test was used to generate a p-value for expression analysis. The prognostic analysis was performed using a Kaplan-Meier curve.

### cBioPortal

cBioPortal (www.cbioportal.org), a comprehensive web resource, can visualize and analyze multidimensional cancer genomics data (14). Five hundred and ninety-four colorectal adenocarcinoma samples (TCGA, PanCancer Atlas) were analyzed. mRNA expression z-scores (RNA Seq V2 RSEM) were obtained using a z-score threshold of ± 2.0. Protein expression z-scores (RPPA) were obtained using a z-score threshold of ± 2.0.

### TIMER

TIMER (https://cistrome.shinyapps.io/timer/) is a comprehensive resource for systematical analysis of immune infiltrates across diverse cancer types and allows users to explore tumor clinical and genomic features comprehensively (15).

### DRUGSURV

DRUGSURV (http://www.bioprofiling.de/GEO/DRUGSURV/) is the first computational tool to estimate the potential effects of a drug using patient survival information derived from clinical cancer expression data sets (16).

### GSEA

GSEA (Gene Set Enrichment Analysis) is a computational method that can determine whether a predefined set of genes shows statistically significant agreement between two biological states (such as phenotype). The difference is used to evaluate the distribution trend of the group of genes in the gene table ranked by the phenotype correlation, to judge its contribution to the phenotype.

### CRN

CRN (Cancer RNA-Seq Nexus) (http://syslab4.nchu.edu.tw/) is the first public database providing phenotype-specific coding-transcript/lncRNA expression profiles and lncRNA regulatory networks in cancer cells. It systematically collected RNA-seq datasets from TCGA, NCBI GEO and SRA (Sequence Read Archive) and resulted in 89 cancer RNA-seq datasets including 325 subsets and 12,167 samples.

### THE HUMAN PROTEIN ATLAS

THE HUMAN PROTEIN ATLAS is a Swedish-based program initiated in 2003 to map all the human proteins in cells, tissues and organs using an integration of various omics technologies, including antibody-based imaging, mass spectrometry-based proteomics, transcriptomics and systems biology (17). In this study, we used THE HUMAN PROTEIN ATLAS database for protein expression profiling.

## Results

### Screening and Identification of DEGs

**Figure 1:**
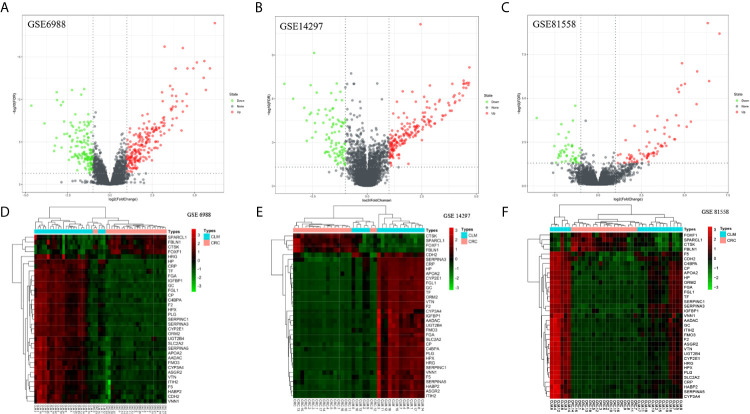
Distributions of differentially expressed genes in CRC and CLM (|log2FC| >1 and adjusted P-value < 0.05) in 63 pairs of matched colorectal primary cancer and liver metastatic tissues. These were volcano maps and heatmaps of 3 data sets, included GSE6988 **(A, D)**, GSE14297 **(B, E)**, and GSE81558 **(C, F)** data set. Red stands for upregulations, green stands for downregulations and black stands for normal expression in volcanoes. Each point represents a gene.

**Figure 2:**
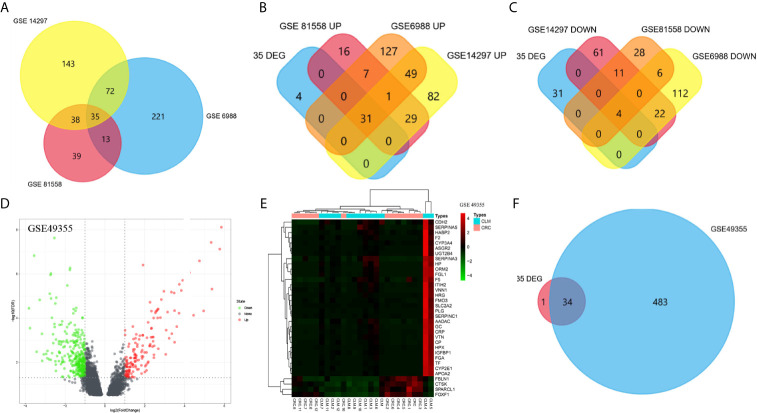
Venn diagram was visualized in FUNRICH software **(A–D)**. The expression profile of GSE49355 were visualized in volcano map and heatmap **(D, E)**. Intersection between GSE49355 and 35 DEGs **(F)**.

We screened microarray datasets from primary CRC and CLM tissue samples from the NCBI-GEO database, which consisted of GSE6988, GSE14297, and GSE81558 datasets. Among them, GSE6988 is based on the GPL4811 platform and was published on February 1, 2008. From this whole-genome dataset of human CLM markers, comprising 123 samples including 25 normal colorectal mucosae, 27 primary CRCs, 13 normal liver tissues, and 27 liver metastases, as well as 20 primary CRC tissues without liver metastases, we selected sample data from 26 pairs of primary CRC with metastatic liver tissues. GSE14297 is based on the GPL6370 platform [Illumina Human-6 v2.0 expression bead chip (extended)] and was published on January 13, 2009. From this primary CRC and related liver metastasis expression spectral dataset, comprising a total of 48 samples including 18 primary CRC, 18 liver metastases, seven normal colorectal mucosa tissues, and five normal liver tissues, we selected data from 18 pairs of CRC and liver metastasis tissues. GSE81558 is based on the GPL15207 platform ([Prime View] Affymetrix) and was published on June 12, 2017. Genomic features of this dataset of liver metastases from CRC patients with expression arrays include a total of 51 samples, including 23 primary CRCs, 19 liver metastases, and nine normal colon mucosal tissues; from this, we selected sample data from 19 pairs of CRC primary cancer and liver metastasis tissues. Then, we used GEO2R to preprocess and filter the original data, using p < 0.05 and [log FC] > 1 as the cut-off criteria, ultimately extracting 315, 233, and 117 differences from these three expression profile datasets, respectively (**Figures 1A–C**). Using FUNRICH software, we identified 35 consistent DEGs from these three genome datasets (**Figure 2A**), including 4 downregulated and 31 upregulated genes (**Table 1** and **Figures 2B, C**). In addition, R software (version 3.6.3) was used to perform cluster analysis and draw a heat map to show the expression of 35 DEGs from the three datasets (**Figures 1D–F**).

**Table 1:** Up and down regulation of 35 differentially expressed genes (DEG) in colorectal cancer liver metastasis.

DEGs	Gene symbol
Up	*HP, CRP, CYP2E1, ORM2, SERPINA3, FGL1, IGFBP1, FGA, F2, GC, APOA2, PLG, HPX, TF, CP, SERPINC1, C4BPA, AADAC, UGT2B4, FMO3, ASGR2, F5, SLC2A2, SERPINA5, CYP3A4, ITIH2, VTN, HABP2, CDH2, VNN1, HRG*
Down	*CTSK, FOXF1, SPARCL1, FBLN1*

To confirm the reliability of DEGs identified from the GSE datasets, we also analyzed the GSE49355 dataset from the GEO database for verification (**Figures 2D, E**). According to the VENN map results using the FUNRICH software, 30 out of 35 DEGs identified during this study were significantly overexpressed in the GSE49355 dataset. Additionally, four genes were also significantly downregulated in the GSE49355 dataset, with only 1 upregulated gene not present in the list of genes (**Figure 2F**). The similarity in expression patterns between upregulated and downregulated genes was 97.14%, which indicated that the candidate genes identified in this study were reliable.

### Gene Ontology (GO) and Signaling Pathway Enrichment Analyses

#### GO Enrichment Analysis

**Figure 3:**
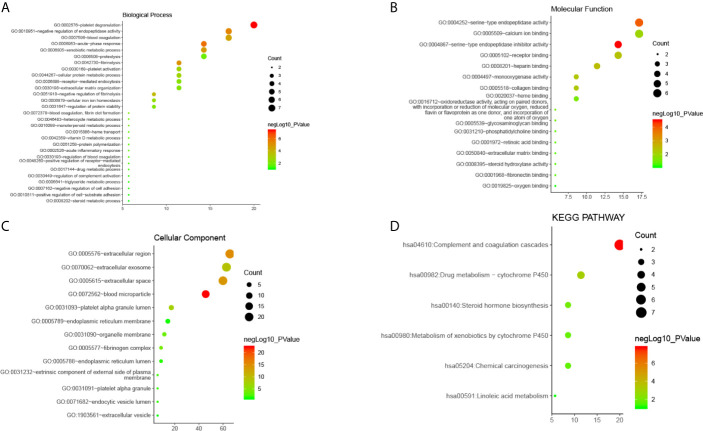
The enrichment analysis of 35 DEGs in CLM (David 6.8). **(A–C)** Bubble diagram of GO enrichment in biological process terms, molecular function terms and cellular component terms. **(D)** Bubble diagram of KEGG enriched terms.

GO analysis from Database for Annotation, Visualization and Integrated Discovery (DAVID) showed that selected candidate DEGs were divided into three functional groups: molecular functional group, biological process group, and cell component group. In the biological process group (**Figure 3A**), DEGs were enriched in many processes such as acute inflammation, post-translational protein modification, platelet degranulation, regulating coagulation and fibrinolytic systems, and regulating protein activation cascades. In the molecular function group (**Figure 3B**), DEGs were mainly enriched in processes that modulate serine endopeptidase inhibitors and hydrolase activity, peptidase modulator activity, glycosaminoglycan binding, heparin and collagen binding, and other processes. In the cell component group (**Figure 3C**), DEGs were mainly enriched in processes that mediate the extracellular space, extracellular region, endoplasmic reticulum, intimal system, platelet α-granules, and cytoplasmic vesicles.

These results indicate that DEGs were mainly enriched in the extracellular area, endoplasmic reticulum and platelet alpha granules, and are mainly involved in inflammation, platelet degranulation, peptidase regulation, protein metabolism, and regulation of the coagulation and fibrinolysis systems.

#### Signaling Pathway Enrichment Analysis

Results from our analysis showed that candidate DEGs shared common signal transduction pathways and reaction processes(**Figure 3D**), including those mainly enriched in the complement-coagulation cascade, drug metabolism (i.e., metabolic enzymes such as cytochrome P450), and steroid hormone synthesis. We also found that these DEGs play a role in the following pathways: chemical carcinogenesis, metabolism of xenobiotics *via* cytochrome P450, linoleic acid metabolism, insulin-like growth factor binding proteins (IGFBPs) that regulate the transport and uptake of insulin-like growth factors (IGFs), post-translational protein phosphorylation, platelet degranulation, activation, aggregation, and other signaling pathways. Among them, the complement-coagulation cascade, platelet activation, degranulation and aggregation, IGFBP-IGF signaling, and drug metabolism were key signal transduction pathways.

### PPI Network Screening and Enrichment Analysis

#### Screening and Modular Analysis of Key Genes

**Figure 4:**
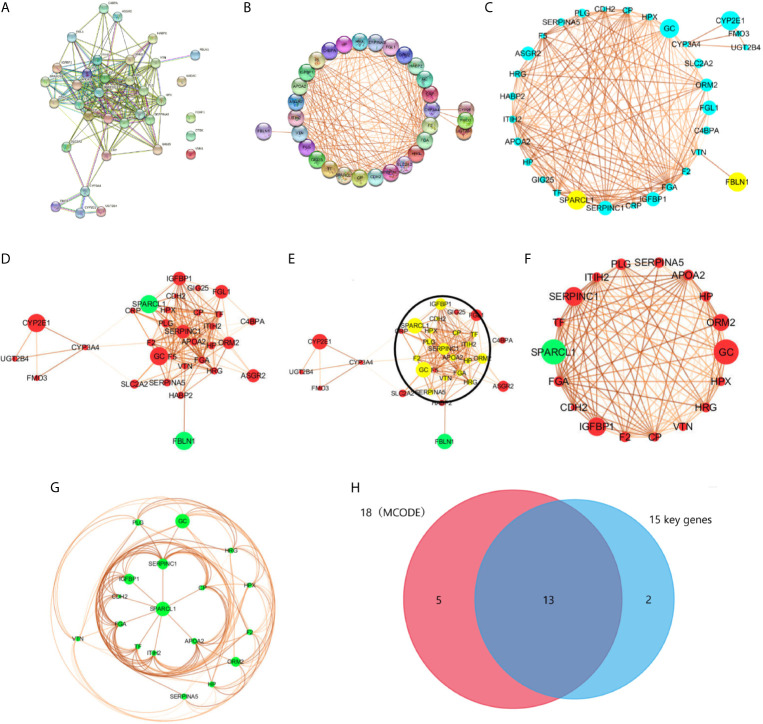
PPI network was visualized in STRING and CYTOSCAPE. **(A)** The PPI network was visualized in STRING that contained 35 nodes and 189 edges, with an average node degree of 10.8, and an average local clustering coefficient of 0.66 with a PPI concentration p-value less than 1.0e-16. **(B)** Four out of 35 DEGs (*AADAC*, *FOXF1*, *CTSK*, and *VNN1*) did not fall within the PPI network. **(C, D)** PPI network of 31 DEGs was visualized in CYTOSCAPE, yellow and green in the node means down regulation, blue and red represents up regulation. The thickness of the node connecting line represents the size of the comparison score. Module analysis of DEGs enrolled in PPI network with the criterion degree cutoff = 2, node score cutoff = 0.2, k-core=2, max depth = 100. The yellow dot in the circle in **(E)** were modular nodes through MCODE analysis. **(F, G)** MCODE Genes were visualized in CYTOSCAPE, included 18 nodes and 119 edges. **(H)** Intersection of 18 modular genes in MCODE and 15 key genes in STRING.

We used the STRING database to filter 35 DEGs into a PPI network containing 35 nodes and 189 edges (**Figure 4A**), with an average node degree of 10.8, and an average local clustering coefficient of 0.66 with a PPI concentration p-value less than 1.0e-16. Among them, four out of 35 DEGs (*AADAC*, *FOXF1*, *CTSK*, and *VNN1*) did not fall within the PPI network; therefore, we ultimately screened 31 DEGs that were designated as key genes. Meanwhile, using k-means clustering analysis, 35 DEGs were divided into three categories, and 26 key genes were selected. Then, we used CYTOSCAPE to remove no-node genes, and make a PPI network diagram based on the interaction and expression between the nodes (**Figures 4B, C**). Using MCODE modular analysis in CYTOSCAPE, 18 candidate genes were screened out (**Figures 4D–H**, **Table 2**).

Based on the PPI network analysis using the STRING database, we divided 26 key DEGs into two modules that included 15 genes. Module 1 comprised proteins that mainly regulate the IGFBP-IGF signaling pathway, whereas module 2 mainly included proteins belonging to the complement-coagulation cascade. Specifically, module 1 included the genes *IGFBP1*, *SPARCL1*, *CDH2*, *ITIH2*, *F5*, *APOA2*, *TF*, *CP*, *FGA*, *SERPINC1*, *F2* and *PLG*. Module 2 included the genes *C4BPA*, *F5*, *FGA*, *SERPINC1*, *F2*, *PLG*, *SERPINA5*and *VTN*. Moreover, *F5*, *FGA*, *SERPINC1*, *F2*, and *PLG* participate in both two pathways. In addition, *FMO3*, *CYP2E1*, *CYP3A4*, and *UGT2B4* were enriched in the drug metabolism-cytochrome P450 signaling pathway. According to the analysis of the three functional groups, 26 DEGs were enriched in extracellular regions, seven DEGs were enriched in platelet alpha particles, and 18 DEGs were enriched in the endoplasmic reticulum.

#### Protein Domain Analysis

In this study, specific protein domains were screened using PPI enrichment analysis, which included Kringle, copper oxidase, trypsin, serine protease inhibitor, hemagglutinin, and cytochrome P450 domains as well as fibrinogen β chain, γ chain, and globular C-terminus domains.

### Gene Set Enrichment Analysis (GSEA) Enrichment Analysis

**Figure 5:**
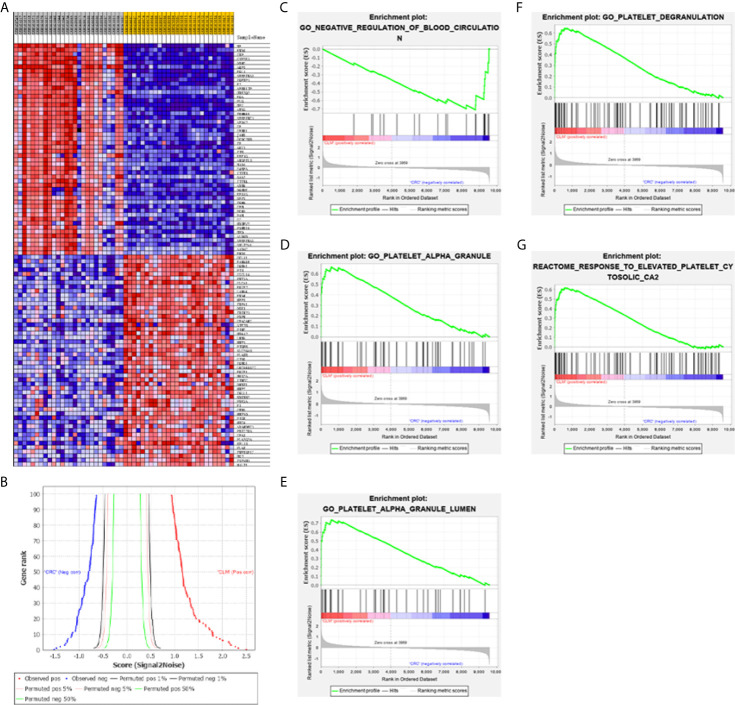
**(A)** GSEA software are used to create a heat map of the top 50 genes with high expression levels in the CLM and CRC phenotypes in GSE6988 (the color range is from “red-blue” to show the range of expression values as “high to low”). **(B)** This figure shows the positive correlation (left) and negative correlation (right) between gene grade and ranking index score. **(C–G)** GSEA analysis about blood circulation and platelet activation-related gene profiles based on CLM and CRC phenotypes in TCGA database.

We used the GSE6988 gene set for enrichment analysis. A total of 9587 effective genes were screened out from the GSE6988 data set, and the genome size filter criterion was set to “minimum equal to 15 and maximum equal to 500”. A total of 8722 gene sets were removed, and the remaining 17002 gene sets were used for enrichment analysis. According to the analysis results, in the CLM phenotype, 6014 gene sets were upregulated; 970 gene sets were significantly enriched under an FDR<25% condition; 440 gene sets were significantly enriched under a p<0.01 condition; and 945 gene sets were significantly enriched under a p<0.05 condition. In the CRC phenotype, 10988 gene sets were upregulated; 17 were enriched under an FDR <25% condition; 219 gene sets were significantly enriched under a p<0.01 condition; 898 gene sets were significantly enriched under a p<0.05 condition. In this study, “|NES|>1, NOM p-val<0.05, and FDR q-val<0.25” were used as the criteria for significant pathway enrichment, and the twenty gene sets with the highest enrichment scores were selected from the CLM and CRC groups (**Figure 5**).

Results show that activation of blood cells and endothelial cells centered on platelets (leading to alterations in cell movement, secretion, enzyme production), Ca2+ metabolism and endocytosis (**Figure 5D**), inflammation, and vascular permeability disruption were enriched in the CLM group. Moreover, 14 of the top 100 genes in the GSEA genetic sequence belong to the 18 DEGs previously screened (**Table 2**).

**Table 2:** 18 DEGs in MCODE modular analysis.

DEGs	Gene symbol
Up	*HPX, CDH2, VTN, IGFBP1, CP, HP, ORM2, APOA2, TF, HRG, PLG, SERPINA5, ITIH2, SERPINC1, FGA, F2, GC*
Down	*SPARCL1*

### Expression Analysis

**Figure 6:**
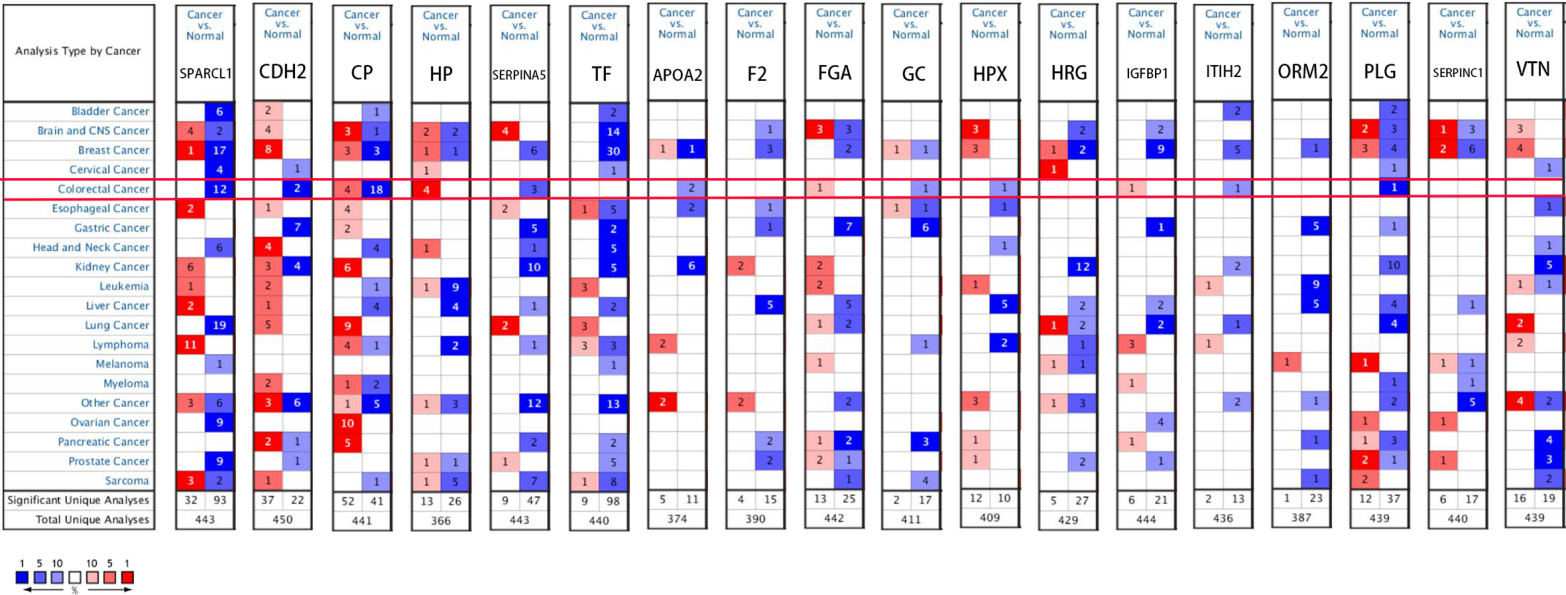
Gene levels of 18 DEGs in CRC (ONCOMINE). The figure shows the numbers of datasets with statistically significant gene over-expression (red) or down-regulated expression (blue) of DEGs.

**Figure 7:**
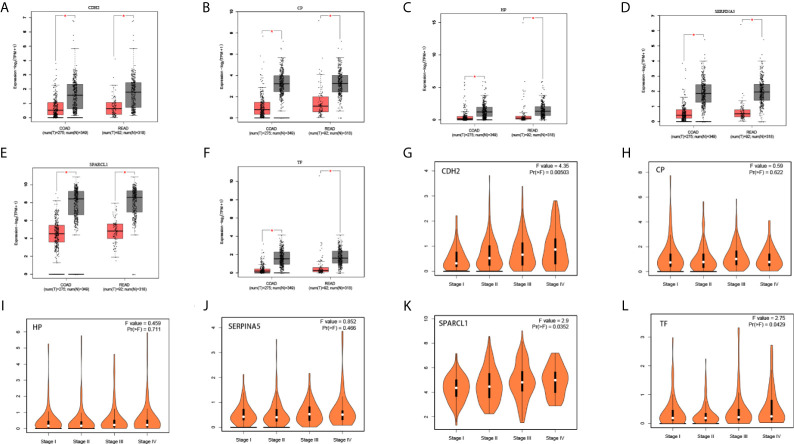
**(A–F)** Differential gene expression analysis of tumor and normal tissues in GEPIA. *SPARCL1*, *CDH2*, *CP*, *HP*, *TF*, *SERPINA5* were significant lowly expressed in cancer tissues. *P < 0.05. **(G–L)** Correlation between six hub genes and the pathological stage of CRC patients (GEPIA).

**Figure 8:**
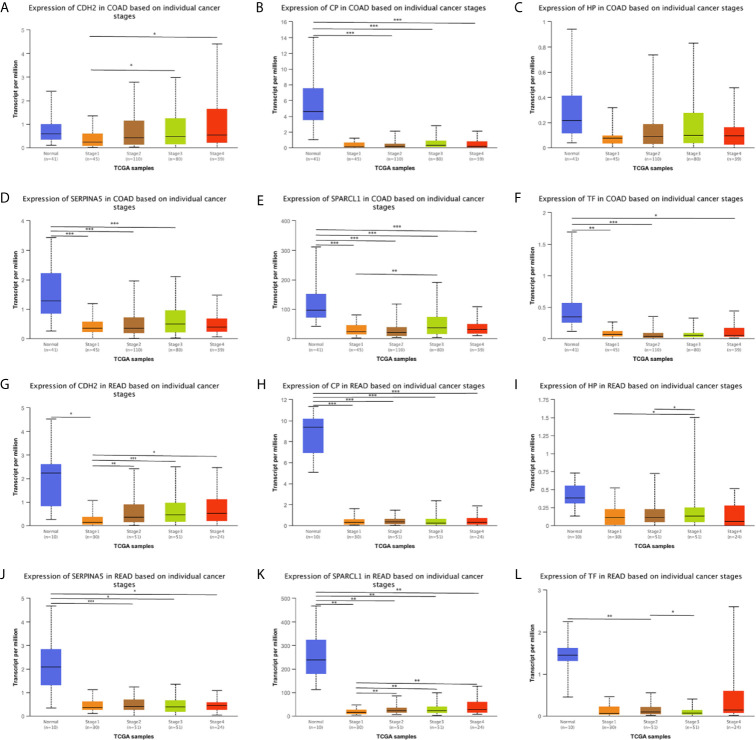
**(A–L)** Expression profile of *SPARCL1*, *CDH2*, *CP*, *HP*, *TF* and *SERPINA5* in subgroups of patients with colon cancer and rectum adenocarcinoma, stratified based on stage criteria (UALCAN). Data are mean ± SE. *P < 0.05; **P < 0.01; ***P < 0.001.

We used the ONCOMINE (**Figure 6**), GEPIA 2 and UALCAN database to analyze the expression of 18 candidate genes in cancer tissues and adjacent normal tissues. Results show that *SPARCL1*, *CDH2*, *CP*, *HP*, *TF* and *SERPINA5* were all significant downregulated in cancer tissues (p < 0.05; **Figures 7A–F**). Moreover, *CDH2, SPARCL1* and *TF* were significantly differentially expressed during the pathological stage in CRC (p < 0.05; **Figures 7G–L**). Additionally, the expressions of *SPARCL1*, *CP* and *TF* differed significantly between stage IV colon cancer and normal (p < 0.05; **Figures 8A–F**), while that of *SPARCL1*, *CDH2*, *CP* and *SERPINA5* differed significantly between rectum adenocarcinoma and normal tissues (**Figures 8G–L**).

### Prognostic Analysis and Correlation Analysis

**Figure 9:**
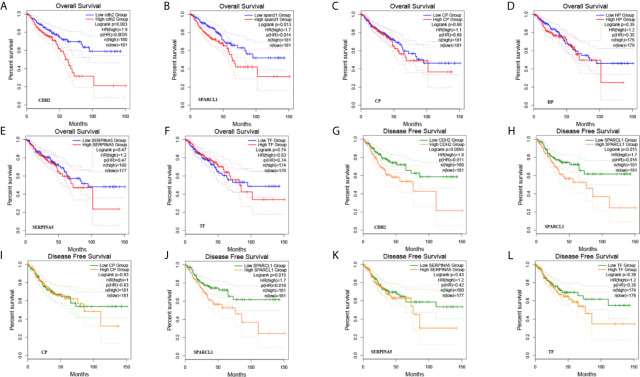
**(A–L)** The prognostic value of DEGs in CRC patients in the overall survival (OS) and disease free survival (DFS) curve (GEPIA).

**Figure 10:**
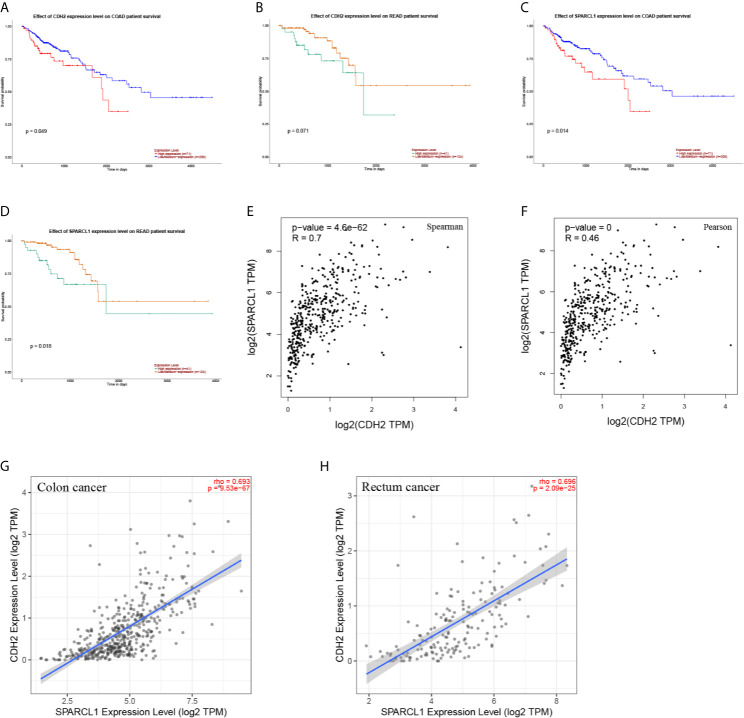
**(A–D)** The prognostic value of *CDH2* and *SPARCL1* patients in the survival curve (UALCAN). **(E, F)** The correlation analysis of *CDH2* and *SPARCL1* in CRC patients in the scatter diagram based on correlation coefficient of Spearman and Pearson (GEPIA). **(G, H)** The correlation analysis of *CDH2* and *SPARCL1* expression level in CRC (TIMER).

A total of six genes were identified as candidate biomarkers. The results of the survival curve analysis indicated that there were two DEGs (*CDH2* and *SPARCL1*) were regarded as prognostic factors (p<0.05). Moreover, CDH2 and SPARCL1 expression levels were found to be significantly related to prognostication. Meanwhile, based on combined Kaplan–Meier curves with log-rank p test, *CDH2* was obviously associated with overall survival (OS; p<0.01) and disease-free survival (DFS; p<0.01) in CRC, with a particularly strong associated observed for rectum cancer (**Figures 9**, **10A–D**). Furthermore, a strong correlation was observed between the expression of *CDH2* and *SPARCL1* (**Figures 10E–H**).

**Figure 11:**
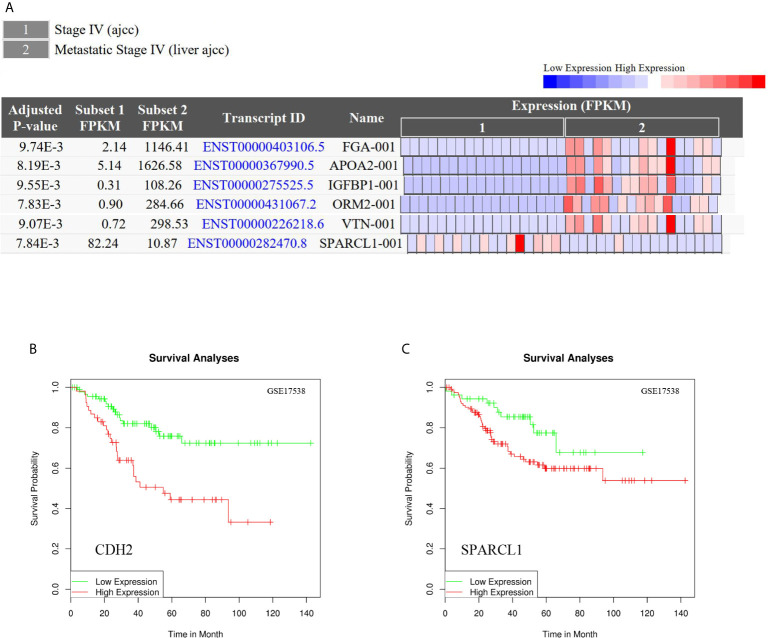
**(A)** Differential gene expression analysis of CRC and CLM in CRN.*SPARCL1, ORM2, IGFBP1, FGA, APOA2*, and *VTN* were significant differential expressed in CLM. (adjusted p-value < 0.01). **(B, C)** CDH2 and SPARCL1 were associated with poor prognosis in CRC in DRUGSURV.

To reveal whether the hub genes identified using the Cancer Genome Atlas (TCGA) database exhibit equal prognostic value in other CRC cases, we used the GSE17538 dataset and GSE50760 as a validation set. *HPX, CDH2, VTN, IGFBP1, CP, HP, ORM2, APOA2, TF, HRG, PLG, SERPINA5, ITIH2, SERPINC1, FGA, F2* and *GC* were upregulated in CLM samples. Meanwhile, *SPARCL1, ORM2, IGFBP1, FGA, APOA2*, and *VTN* were significantly differentially expressed (adjusted p-value < 0.01). Further, *CDH2* and *SPARCL1* were associated with poor prognosis in CRC, suggesting that both may represent potential genetic biomarkers for poor prognosis in CRC, and may provide potential value for CRC treatment in the future (**Figure 11**).

We also observed CDH2, CP, HP, TF, and SERPINA5 were upregulated in liver metastatic cancer tissues and downregulated in primary cancer tissues; whereas SPARCL1 exhibited the opposite expression pattern. Moreover, compared to CRC tissues, SPARCL1, CDH2, CP, HP, TF, and SERPINA5 were relatively highly expressed in normal colorectal tissues. In addition, compared to liver metastatic cancer tissues, CP, HP, TF, and SERPINA5 were upregulated in normal liver tissues, while the expression CDH2 and SPARCL1 did not exhibit significant differences between the two tissues. Therefore, in the observed tissues, SPARCL1 was most highly expressed in normal colorectal tissues, and CDH2 had the highest expression in normal liver tissues and liver metastatic cancer tissues, while CP, HP, TF, and SERPINA5 with the most highly expressed in normal liver tissues.

### Genetic Alteration and Co-Expression Analyses

**Figure 12:**
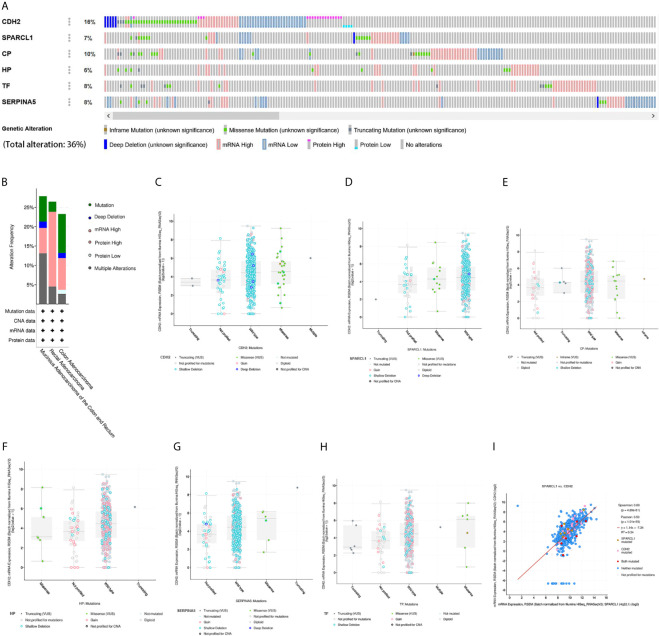
Genetic alteration and co-expression analyses of six hub genes (*SPARCL1*, *CDH2*, *CP*, *HP*, *TF* and *SERPINA5*) in CRC patients. **(A)** Summary of alterations in six hub genes in CRC. **(B)** Summary of mutation type in CRC. **(C–H)** Mutation type and copy number of six hub genes. **(I)** The correlation analysis of mutation in mRNA expression between *CDH2* and *SPARCL1* in CRC.

We then performed a comprehensive analysis of the molecular characteristics of six hub genes (CDH2, SPARCL1, CP, HP, TF, and SERPINA5) using the Pan Cancer Atlas of TCGA. Results show that CDH2, SPARCL1, CP, HP, TF, and SERPINA5 were altered in 16%, 7%, 10%, 6%, 8%, and 8% of the queried CRC samples, respectively. Additionally, the six hub genes were altered in 213 (36%) queried samples. Enhanced mRNA expression was the most commonly observed change in these samples. We next explored the potential co-expression of these hub genes and found that the expression of *CDH2, SPARCL1, CP, HP, TF* and *SERPINA5* exhibited significant correlations, with the strongest association observed between *CDH2* and *SPARCL1* (**Figure 12**).

### Prognostic Gene Validation Using Clinical Tissue Samples

**Figure 13:**
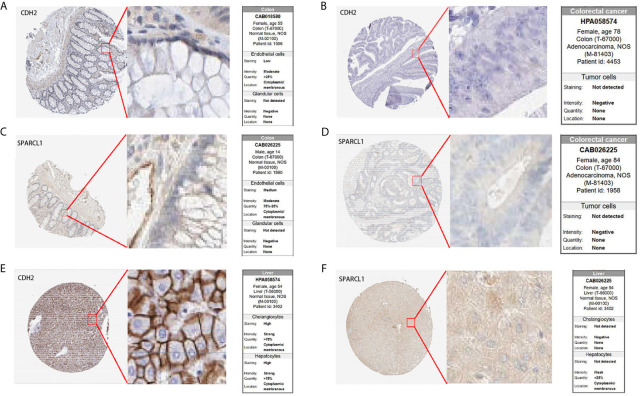
IHC analysis of *CDH2* and *SPARCL1* with prognostic values. **(A–F)** Differentially expressed proteins of *CDH2* and *SPARCL1* with prognostic values in CRC, colorectal normal tissues and liver normal tissues in The Human Protein Atlas database.

To further confirm the prognostic value of the hub genes with prognostic values, we used immunohistochemical (IHC) staining to detect the protein expression of CDH2 and SPARCL1 in normal tissues and tumor tissues. The results showed that compared to normal tissues, CDH2 and SPARCL1 were significantly under-expressed in primary CRC tissues. Meanwhile, SPARCL1 was relatively overexpressed in normal colorectal tissues, and CDH2 was highly expressed in normal liver tissues (**Figure 13**) which agreed with our research conclusions.

## Discussion

In the past few decades, many basic and clinical studies have revealed causes and potential mechanisms that mediate CRC formation and development; however, the incidence and mortality of this disease are very high, and the high recurrence rate and liver metastasis are the main contributors to patient mortality (18, 19). Most studies have focused on a single genetic event or results derived from a single cohort study (20). However, approximately 90% of CRC cases develop sporadically, and only a few (less than 10%) can be attributed to genetic sources (21). With the widespread application of gene-related technologies such as gene chips and NGS, a large amount of core slice data has been generated, and most of the data have been stored in public databases. Therefore, integrating and re-analyzing these datasets can provide valuable clues for new research.

In recent years, researchers have conducted many microarray data analysis studies on CRC (22) and have obtained hundreds of DEGs, but the analysis of microarray data from CLM is lacking. Due to the organization of independent research or sample heterogeneity, the results are always limited or inconsistent, and reliable and effective biomarkers have not been identified. Moreover, most NGS studies have not dealt with the functional interpretation of these DEGs to clearly identify suitable key genes. The combination of integrated bioinformatics methods and expression profiling techniques will likely solve this shortcoming. In the present study, we integrated three cohort datasets from different sources and conducted an in-depth analysis of these data using multiple bioinformatic methods. Moreover, to avoid the potential impact of radiotherapy and chemotherapy on the genomic clonal changes in tumor tissues, we sought to exclude any such samples. Comparative gene expression profiling of matched CRC and CLM tissues revealed very few statistically significant, DEGs. Nevertheless, despite the high similarity at the genomic level, we were able to detect these subtle changes. Accordingly, two pathways, 18 DEGs and 6 hub genes were screened based on genome and transcriptome sequencing data, as well as the analysis of expression profile and prognosis.

For decades, researchers have noticed a certain relationship between the different components of the insulin-like growth factor IGF system and the development of cancers such as solid tumors and hematological malignancies (23–26). As polypeptide growth factors, IGF is a key regulator of different cancer progression stages and is related to tumorigenesis, development, and metastasis (23, 24, 27). The IGF system is complex; in addition to insulin, it also includes two IGFs (IGF1 and IGF2) and their surface receptors, six IGFBPs, IGFBP protease, and insulin substrate proteins (IRS1–6). The production and secretion of different members of the IGF family during the development of colon cancer are affected by genetic and environmental factors (28–30). IGF1 and IGF2 are highly expressed in CRC, and a variety of metabolic disorders such as obesity, dyslipidemia, hyperinsulinemia, and glucose homeostasis are common in these patients, making the IGF system a biomarker of human CRC susceptibility and prognosis (31–34). Recent data also describe the CRIS-D subtype, which includes the activation of the wingless/integration (Wnt) pathway and the overexpression and amplification of IGF2. An analysis of approximately 300 publicly available CRC datasets indicated that patients with CRIS-D tumors have longer disease-free survival (35). CRC is also called obesity-related cancer, and its pathogenesis is related to being overweight and obese, caused by PI3K/AKT pathway activation (36, 37). Insulin and IGF signaling combined with chronic inflammation are also important factors for obesity, promoting CRC development (28).

In all fetal tissues, *IGF2* is transcribed from three ubiquitous promoters (P2–P4). In the adult liver, *IGF2* gene transcription is initiated by the liver-specific promoter (P1), but the P2–P4 promoters are still active in adult peripheral tissues (29). Studies suggest that IGF2 has an autocrine effect (30, 38–40). Compared to that in primary CRC, the expression level of IGF2 in CRC metastatic tumors (mainly liver metastases) changes to a greater extent, and its expression can be both high (41) and low (42). IGF2, together with transforming growth factor-α and matrix metalloproteinase-2, is used as a tumor staging marker (43, 44) and is also a key factor in the early stage of CRC (45).

The autocrine/paracrine effect of IGF2 mainly involves polypeptides produced by extrahepatic tissues, including colorectal tumor cells and CRC tumor stromal cells (cancer-associated fibroblasts) (46, 47). IGF2 is involved in the induction of some activation and suppression markers (e.g. Wnt5a, CEACAM6, IGFBP3, KPN2A, BRCA2, and CDK1) (48). Studies have shown that IGF2 concentrations are positively correlated with more advanced CRC (49, 50), and this correlation is positively correlated with an increase in disease stage and regional lymph node metastasis (51). The general expression/overexpression of IGF2 in primary CRC and liver metastases is usually associated with disease progression, increased grade or stage, or poor survival and prognosis (52, 53).

At present, the role of IGF2 in the metastasis of CRC is still unclear. Studies conducted on highly metastatic CRC cell lines indicate that IGF1 and IGFBP1, but not IGF2, are potentially associated with CRC metastasis (54). In addition, there are some studies that suggest that IGF2 is an important tissue marker for tumor progression in patients with liver metastases from CRC (41, 43). The PPI network analysis results of the present study indicate that *IGFBP1*, *SPARCL1*, *CDH2*, *ITIH2*, *F5*, *APOA2*, *TF*, *CP*, *FGA*, *SERPINC1*, *F2*, and *PLG* participate in IGFBP processes to regulate IGF transport and uptake signaling pathways, and these DEGs might participate in this pathway to affect the invasion and metastasis of CRC. Meanwhile, *SPARCL1*, *CDH2*, *CP* and *TF* may play a key role. Moreover, *CDH2*, *CP* and *TF* were low expressed in primary cancer than normal tissues, but highly expressed in metastatic liver cancer. Therefore, these three genes might have the key effect of promoting cancer metastasis.

In the GO analysis results of the present study, DEGs were found to be mainly enriched in extracellular regions, vacuole cavities, and platelet alpha particles in cell component modules and to participate in signal transduction pathways such as the complement-coagulation cascade. The main function of platelets is to recognize blood vessel damage and promote thrombosis, thereby stopping bleeding. However, this characteristic of platelets also contributes to the cancer development, which can interact with circulating tumor cells (CTCs). Receptor recognition and factor-mediated interactions between tumor cells and platelets stimulate platelet activation, factor release, and aggregation, thus promoting tumor cell survival and cancer progression. Tumors are essentially composed of multiple heterogeneous molecules and sub-cytokines, which can be exchanged with the circulatory system through various mechanisms. Compared to a discrete tissue biopsy, a liquid biopsy method that collects relevant lesion components in the vascular system by draining tumor cells might be more conducive to the discovery of a more representative tumor microenvironment (55).

In recent years, the role of platelets in tumor metastasis has become increasingly important. Studies have reported that increased platelet counts can promote ovarian cancer (56). Circulating platelets are derived from megakaryocytes in the bone marrow. Platelets have a special function in the highly organized and progressive response to vascular injury. They are activated, releasing special particles and forming aggregates, generating coagulation plugs, and controlling bleeding (57). In addition, the transcriptome of platelets is unique and was proven to be different from that of other cell types. The number of transcriptomes of platelets is estimated to be approximately 3,000-6,000 (58, 59). Further, there is increasing evidence that the progression of cancer might be the result of platelet-related diseases. Platelet microparticles (PMPs) are thought to transfer receptors to the surface of tumor cells through membrane fusion, induce tumor cell chemotaxis, promote tumor cell proliferation, and induce the expression of IL-8, MMP-9, and VEGF (60). Other studies have also shown that PMPs can increase metastasis and angiogenesis, and prevent apoptosis (61–63). CTCs are easily killed by the sheer force of blood flow and natural killer cells, which makes it difficult for tumor cells to initiate the metastatic cascade. Some studies have pointed out that the activation of the coagulation cascade with the activation of platelets, mediated by tumor cell tissue factors, can protect circulating CTCs by wrapping them in a platelet-rich thrombus (51, 64), resulting in immune escape. In addition, platelets can promote CTC migration by preventing CTCs from adhering to the endothelial cells of the vessel wall (51). One receptor–ligand pair with this function is ADMA9 on tumor cells, which binds integrin α6β1 on the surface of platelets. This interaction is thought to promote platelet activation, granule secretion, and tumor cell migration through the endothelium (65).

Platelet-secreted proteins, such as agrin and thrombin-reactive protein 1, can also enhance the activity and expression of MMP-9 through the p38MAPK pathway, thereby stimulating the aggressiveness of colon cancer (66). In addition, platelets can also induce the transformation of epithelial cells to mesenchymal cells by transforming growth factor-β and nuclear factor-κB signals in tumor cells. Studies have revealed that high platelet counts are significantly associated with invasion, metastasis, and a reduced survival rate in CRC patients (67, 68). Activated platelets increase phosphatidylserine exposure on their surface, which might put them in a more favorable state of coagulation in CRC patients. In addition, the liver has a double blood supply, and approximately two-thirds to three-quarters of the blood supply comes from the portal vein, with the rest originating from the hepatic artery. Gastrointestinal tumor cells can spread to the liver through portal vein circulation and systemic circulation (69). In this path, the coagulation state is more conducive to the formation of cancerous tumor cells and arrival in liver tissue.

The PPI network analysis results of the present study showed that C4BPA, F5, FGA, SERPINC1, F2, PLG, SERPINA5, and VTN are enriched in the complement-coagulation cascade signaling pathway, with F5, FGA, ORM2, HRG, PLG, SERPINA5, and GIG25 enriched in platelet alpha particles. Among them, F5, FGA, PLG, and SERPINA5 were found to be both enriched in the signaling pathway of the complement and coagulation cascade and platelet alpha particles. Therefore, these DEGs might shape the microenvironment of vascular tumors through the platelet coagulation cascade, thus participating in the occurrence and development of CLM.

According to bioinformatics analysis results, we suggest that these candidate DEGs might be tissue-specific and mainly expressed in liver tissues. However, based on the verification of the TCGA database it was determined that although the candidate DEGs are enriched in the hepatobiliary system, the difference in expression levels between normal tissues and cancer tissues was not significant. Thus, it can be speculated that the difference in the expression of these genes might not only be caused by primary cancer of the hepatobiliary system. The occurrence of metastatic liver cancer is most likely related to the driving effect of DEGs in the primary foci of CRC, resulting in changes in liver tissue gene sequences and expression profiles. This driving effect might require the help of the tumor microenvironment in extracellular fluid. A small number of DEGs or their protein products in primary CRC might settle in liver tissue along with body fluids (such as blood and lymph) to promote the development of metastatic cancer. These protein products might play key roles based on the following functional domains: Kringle, trypsin, copper oxidase, serine protease inhibitors, and hemagglutinin. In addition, the tumor metastasis induction produced by the candidate DEGs might not be caused by the expression of a single independent gene but rather by multiple genes or a gene expression complex acting together. The genetic ontology results showed that the candidate DEGs we selected are mainly derived from extracellular regions and platelet alpha particles, which further validates our perspective. In our results of GSEA, in the CLM group, there was a high likelihood that blood cells, such as platelets were activated, thus promoting particle secretion (endocytosis), tumor cell metastasis, transport of particles in the blood in the form of phospholipid microvesicles, and circulation through blood and lymph transfer to the liver to complete the migration. Tumor cell particles could also activate vascular endothelial cells, thus affecting vascular permeability and enabling migration outside the blood vessel. In addition, Ca2+ plays a key role in regulating platelet secretion.

Certain limitations were noted in our study. For instance, the analysis of genome and transcriptome data alone cannot fully reflect the specific molecular mechanism of liver metastasis in CRC, and the variation of each gene is highly uncertain. Moreover, additional independent cohort, *in vitro* and *in vivo* studies are needed to validate our results. Nevertheless, our results provide a reliable theoretical basis for additional research to elucidate the specific mechanism underlying the malignant progression of CRC. Moreover, the identified candidate DEGs are primarily involved in the complement-coagulation cascade and IGFBP-IGF signaling pathways, which play an important role in shaping the microenvironment of vascular tumors. These findings significantly improve the understanding of the cause and underlying molecular events in CLM and provide potentially reliable biomarker information for early detection and diagnosis of CLM, as well as the candidate genes and pathways that could be used as therapeutic targets.

We also found that *SPARCL1* was downregulated in liver metastasis, which may have been caused by the loss of various activities such as platelet activation driving the acquisition of a metastatic signature; hence, *SPARCL1* may have been lost as a normal liver-specific gene. Similarly, *CDH2*, *CP, HP, TF*, and *SERPINA5* may gradually transform into normal liver tissue-specific genes during liver metastasis. Moreover, *CDH2* exhibited the highest mutation frequency, while its expression did not differ between normal liver and metastatic liver cancer tissues. Hence, *CDH2* and *SPARCL1* likely play a more significant role in the metastasis of primary CRC cells to normal liver tissues.

*CDH2* (Cadherin 2) encodes N-cadherin, a classical cadherin superfamily member, and is associated with neural crest differentiation, mesenchymal stem cells, and lineage-specific markers, according to the GeneCards database. We observed that CDH2 was enriched in the endoplasmic reticulum, plasma membrane, and cell junctions and may participate in the IGFBP processes to regulate IGF transport and uptake signaling pathway to affect the invasion and metastasis of CRC. Moreover, CDH2 overexpression likely shifts the hierarchy of stem and progenitor cells within liver metastases, resulting in enhanced self-renewal, and potentially affecting the clinical behavior of CRC. In addition, Ca2+ plays a key role in regulating platelet secretion. As a calcium-dependent cell adhesion protein, CDH2 can mediate homotypic cell-cell adhesion by dimerization with a CDH2 chain from another cell to promote tumor cell metastasis.

*SPARCL1* (SPARC-like 1) is associated with calcium ion binding according to the GeneCards database. We observed that *SPARCL1* was enriched in the extracellular region and might participate in the IGFBP-IGF signaling pathway. Moreover, as a plasma protein, *SPARCL1* may promote tumor cell metastasis by promoting the secretion of granules (endocytosis and exocytosis). In addition, *SPARCL1* and *CDH2* may be related to the activation, secretion, aggregation, and migration of platelets. Therefore, *SPARCL1* and *CDH2* may be co-expressed and work in concert to promote liver metastasis of CRC.

## Conclusion

In conclusion, the complement-coagulation cascade and IGFBP-IGF pathway may be key signaling pathways for CLM. We found that *HPX*, *SPARCL1*, *CDH2*, *VTN*, *IGFBP1*, *CP*, *HP*, *ORM2*, *APOA2*, *TF*, *HRG*, *PLG*, *SERPINA5*, *ITIH2*, *SERPINC1*, *FGA*, *F2* and *GC* were key candidate genes, and *SPARCL1*, *CDH2*, *CP*, *HP*, *TF* and *SERPINA5* play a central role. Moreover, *CDH2* and *SPARCL1* were significantly related to the prognosis of CRC. Identifying these candidate genes and targeting these specific pathways maybe more accurately to diagnose, prevent and treat CRC and CLM.

## Data Availability Statement

The original contributions presented in the study are included in the article/**Supplementary Material**. Further inquiries can be directed to the corresponding author.

## Ethics Statement

The studies involving human participants were reviewed and approved by Department of Gastrointestinal Surgery, The First Affiliated Hospital of Sun Yat-sen University, Guangzhou, People’s Republic of China. Written informed consent for participation was not required for this study in accordance with the national legislation and the institutional requirements.

## Author Contributions

TZ and KY contributed equally to this study, including study concept and design, analysis and interpretation of data, drafting of the manuscript and critical revision of the manuscript for important intellectual content conceived of, and designed the study. YW, MX, SC, and CC performed the literature search, acquisition of data, and statistical analysis. JM supervised the whole study and edited and reviewed the manuscript. All authors contributed to the article and approved the submitted version.

## Funding

This work was supported by the Guangzhou Association for Science & Technology (grant number: K2019010101042, K2019010201082, K2019070201006, K20200201002 and K20210101013), Guangzhou Municipal Science and Technology Bureau (grant number: 202002020048) and Department of Science and Technology of Guangdong Province (grant number: 2020A1414040006).

## Conflict of Interest

The authors declare that the research was conducted in the absence of any commercial or financial relationships that could be construed as a potential conflict of interest.

## Publisher’s Note

All claims expressed in this article are solely those of the authors and do not necessarily represent those of their affiliated organizations, or those of the publisher, the editors and the reviewers. Any product that may be evaluated in this article, or claim that may be made by its manufacturer, is not guaranteed or endorsed by the publisher.

## Abbreviations

AI, Artificial Intelligence; CLM, Colorectal Cancer Liver Metastasis; COAD, Colon Adenocarcinoma; CRC, Colorectal Cancer; DEG, Differentially Expressed Genes; DFS, Disease-Free Survival; GEO, Gene Expression Omnibus; GO, Gene Ontology; GSEA, Gene Set Enrichment Analysis; IGF, Insulin-like Growth Factor; IGFBPs, Insulin-like Growth Factor-Binding Protein 1; KEGG, Kyoto Encyclopedia of Genes and Genomes; LIHC, Liver Hepatocellular Carcinoma; NGS, Next Generation Sequence; OS, Overall Survival; PPI, Protein-protein Interaction Network; READ, Rectal Adenocarcinoma; TCGA, The Cancer Genome Atlas; TME, Tumor Microenvironment; TIMER, Tumor Immune Estimation Resource.
